# Vertical gingival display changes associated with upper premolars extraction orthodontic treatment: A prospective clinical trial

**DOI:** 10.4317/jced.57538

**Published:** 2020-11-01

**Authors:** Michel F. Fallas, Elham S. Abu-Alhaija, Susan N. Alkhateeb, Shadi S. Samawi

**Affiliations:** 1 Master student, Division of Orthodontics, Department of Preventive Dentistry, Faculty of Dentistry, Jordan University of Science and Technology, P.O. Box 3030, Irbid-Jordan; 2 Professor, Division of Orthodontics, Department of Preventive Dentistry, Faculty of Dentistry, Jordan University of Science and Technology, P.O. Box 3030, Irbid-Jordan; 3 Professor, Division of Orthodontics, Department of Preventive Dentistry, Faculty of Dentistry, Jordan University of Science and Technology, P.O. Box 3030, Irbid-Jordan; 4 Private orthodontic practice, Amman – Jordan

## Abstract

**Background:**

Extraction of upper bicuspids have been anecdotally blamed to increase the vertical gingival display (VGD) anteriorly. However, the extraction may be needed in some cases in order to correct the underlying orthodontic problem. Objectives: To investigate and compare vertical gingival display (VGD) changes associated with upper (first vs second) premolars extraction during orthodontic treatment.

**Material and Methods:**

Design: A prospective clinical trial. Setting: Postgraduate dental teaching clinics at Jordan University of Science and Technology (JUST). Sample population: Sixty orthodontic patients were included in the study. They were treated with upper first or second premolars extraction according to the underlying problem and the individualized treatment plan of each patient. Records (radiographs, study casts and clinical photographs) were taken for all subjects pre- and post- orthodontic treatment. Outcome measures: Pre- and post-treatment VGD, lip length in static and dynamic positions and the amount of upper teeth retractions were recorded. The paired and the independent t- test were used to detect differences within/between groups. Factors affecting VGD were investigated using backward stepwise linear regression analysis.

**Results:**

In both static and dynamic captures, VGD increased after orthodontic treatment in both premolars extraction groups. Pre- and post-treatment variables differed significantly in groups 1 and 2. VGD changes were similar in both treatment groups. A significant association was found between VGD change during orthodontic treatment and upper canine retraction (*P*<0.001), pre-treatment ANB angle (*P*<0.01) and upper incisor retraction(*P*<0.05).

**Conclusions:**

The amount of anterior VGD increases after upper premolars extraction. The increase in VGD after first and second premolars extractions was comparable. The increase in VGD after orthodontic treatment is associated with the amount of canine retraction, pre-treatment ANB and the amount of incisor retraction.

** Key words:**Vertical gingival display, tooth extraction, dental esthetics.

## Introduction

There is general consensus regarding an attractive smile, which occurs when little gingiva is displayed upon smiling (1-3). A gummy smile (GS) is diagnosed when there is excessive vertical gingival display (VGD) between the lower border of the upper lip and the free gingival margin of the upper anterior teeth (4). It has been confirmed that the teeth size, amount of incisal show and the position of upper lip are important characteristics in self-recognition of smile attractiveness and more importantly, the VGD (1). Studies have indicated different threshold levels of VGD that can be perceived as acceptable (1-3), beyond these levels, the VGD will adversely affects the perceived beauty of a smile.

Kokich *et al.* (2) found that VGD during smiling was not generally noticeable by general dental practitioners or laypeople unless it was 4mm at least and Van der Geld *et al.* (1) suggested that patients with 2mm to 4mm of VGD were considered esthetically accepTable while Abu Alhaija *et al.* (3) reported that general dental practitioners, orthodontists and laypeople considered a VGD of 2mm or more as unattractive.

Anecdotally, extraction of teeth has been attributed to increase the VGD. However, extraction of upper premolar may be needed in some patients with mild gummy smile in order to correct the underlying orthodontic problem. To our knowledge, no study to investigate the effect of upper first or second premolar extraction on VGD in orthodontically treated subjects is said to exist. The objectives of this study were to record and compare changes in the VGD in subjects treated with fixed appliance with upper premolars (first and second) extraction treatment plan.

## Material and Methods

-Trial desing

This study was a parallel group prospective clinical trial

-Subjects and Methods

Ethical approval for this study was obtained from the Institutional review board (IRB)/ Jordan University of Science and Technology /JUST(IRB No. 86/117/2018). The participants for this study were recruited from patients attending postgraduate orthodontic clinics/ JUST The inclusion criteria were as follows: age 17 years or more, skeletal class I or class II malocclusion, upper premolars extraction treatment plan, no previous orthodontic treatment. The exclusion criteria were poor oral hygiene, lower arch extraction and smoking. A written informed consent was attained from all participants before orthodontic treatment.

-Sample size calculation revealed that for a 90% power and 5% precision and assuming an overall attrition rate of 10%, initial recruitment should target a total of 20 patients per group. Sample size was calculated (5) as follows: N=((r+1)(Zα/2+Z1-ß)2 δ2)/rd2 Where Zα/2 is the normal deviate at a level of significance (Zα/2 is 1.96 for 5% level of significance) and Z1-β is the normal deviate at 1-β% power with β% of type II error (1.28 at 90% statistical power), r= n1/n2 is the ratio of sample size required for 2 groups, generally it is one for keeping equal sample size for 2 groups, δ(0.92) is obtained from a previous study (3) and is the expected difference between the 2 groups (1mm).

Sixty orthodontic patients were included in the study. They were treated with extraction of upper first or second premolars according to the underlying problem and the individualized treatment plan of each patient. The pretreatment baseline cephalometric measurements for the investigated groups are presented in Table 1.

**Table 1:**
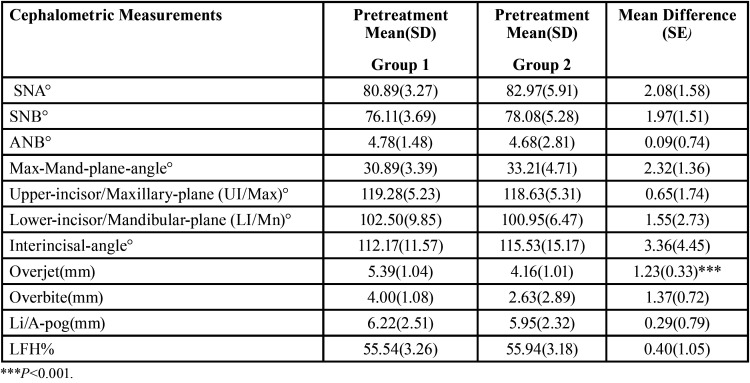
Means, SD for the baseline cephalometric measurements, difference between means and SE between the 2 studied groups.

**Figure 1:**
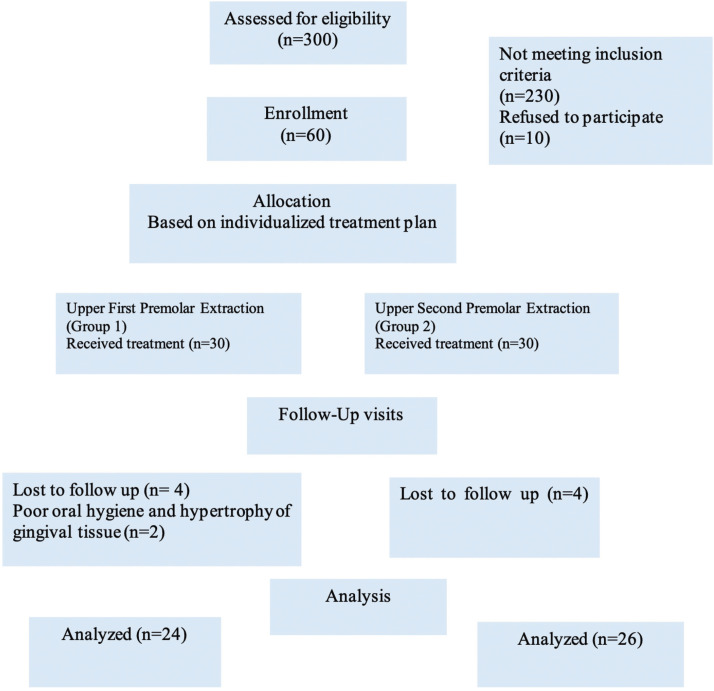
Flowchart showing patient flow during the trial.

At the time of final records taking and analysis, 6 patients from group 1 and 4 patients from group 2 were excluded (missing appointments and poor oral hygiene) (Fig. 1).

Subjects were allocated into one of 2 groups as follows: -

Group 1: - Upper first premolars extraction 

 Included 24 patients (7 males,17 females) with a mean age of 21.56±3.19yrs who were treated with fixed orthodontic appliance for 2.22±0.3yrs.

Group 2: - Upper second premolars extraction 

Included 26 patients (8 males,18 females) with a mean age of 22.16±3.59yrs who were treated with fixed orthodontic appliance for 2.25±0.30yrs.

Pre- and post- treatment records (orthopantomogram, lateral cephalogram, study casts, clinical photographs, digital video recording) were taken for all subjects.

All patients were treated using pre-adjusted edgewise fixed appliance (3M-Gemini-Uniteks, 0.022-inch MBT-prescription brackets) by the same orthodontic postgraduate student (M.F.). Brackets were placed at the same height (midfacial-axis) for the upper anterior teeth in all subjects. Patients were followed-up monthly. Initial dental alignment started with 0.014-inch Nickle Titanium (NiTi) archwire then with a sequence of 0.016-inch, 0.018-inch, 0.016X0.022-inch, 0.019X0.025-inch NiTi archwires before 0.019X0.025-inch stainless steel (SS) rectangular archwires were inserted. Upper space closure was carried out using elastic power chain for all patients. No upper arch extrusive or intrusive mechanics were used throughout the study (intermaxillary elastics, curve of Spee, utility arch and miniscrews in the upper arch).

-Clinical photographs and video recording 

The clinical photographs and video records were taken with a digital video camera (Canon EOS-70D). Five seconds of video, yielding 150 frames was taken for each patient. The frontal photographs and videos were recorded in standardized fashion with the camera at a fixed distance from the patient (1.5m). The patient’s head was placed in a cephalometric head holder to obtain natural head position and the patient was asked to rehearse the phrase “Chelsea eats cheesecake on the Chesapeake” and then to smile. The video was downloaded to the computer and the frame that best represents the patient’s natural unstrained social smile was selected. Table 2 shows definition of the measurements calculated using the static and video captures.

**Table 2:**
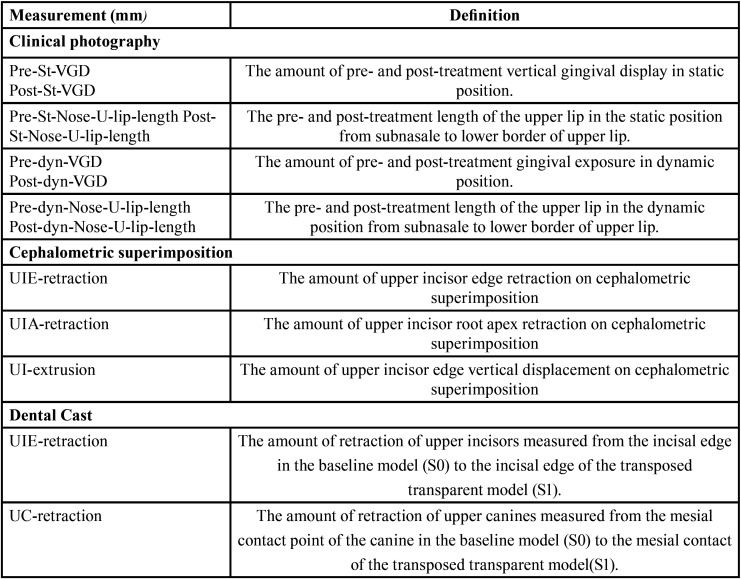
Definition of measurements used in the study.

-Cephalometric superimposition (Table 2)

The pre-treatment maxillary incisors tracing was placed on the graphic Tablet of the digitizing system over a millimeter graded sheet. Post-treatment maxillary incisors were traced on the pre-treatment cephalogram. The difference between every related point was measured by calculating the number of squares (each square on the graded sheet equal 1 mm).

-Dental cast measurements (Table 2)

Alginate impressions were taken before and after treatment and study casts were fabricated and scanned with a Ceramill Map 400- scanner with accuracy of 0.02 mm (AmannGirrbach, Koblach, Austria) to obtain a 3-dimensional (3D) model. By using Ceramill Mind design (CAD; computer-aided design) software of AmannGirrbach Company, 3D model measurements were obtained. The accuracy of measurements was performed by calibration of the program each week.

The baseline model (S0) was superimposed to the post-treatment model (S1) to determine the amount of retraction of anterior teeth and the amount of molar protraction. The reference landmark used was the rugae area as recommended by previous researchers (6-8).

-Primary outcome

• VGD:- It was measured from the lower edge of the upper lip to the gingival margins of the incisors and canines. This was measured pre- and post-orthodontic treatment for both static and dynamic lip positions.

• Upper lip length: - It was measured from subnasale to lower border of upper lip. This was measured pre- and post-orthodontic treatment for both static and dynamic lip position.

-Secondary outcome

• Upper Anterior teeth retraction: It was calculated as the horizontal distance between pre-t and post-treatment incisal edges.

• Upper incisors extrusion: It was calculated as the vertical distance between pre- and post- treatment incisal edges

The records of 10 subjects were randomly selected and measurements were done twice with 2-week interval. The Dahlberg formula was used to calculate the standard error of the method. Dahlberg errors ranged from 0.01mm for St-VGD to 0.32mm for Li/A-Pog and from 0.30° for ANB to 1.03° for Ui/Max. 

-Statistical analysis

Statistical analysis was performed using the Statistical Package for the Social Sciences computer software (SPSS 22, SPSS Inc., IL, USA). Intention to treat (ITT) analysis was applied. Paired t-test was conducted to examine and define the differences between the studied variables at the different time intervals before and after orthodontic treatment. Independent t-test was carried out to detect the differences between the 2 studied groups. Backward stepwise linear regression analysis was used to determine the effects of the studied variables on the amount of VGD after extraction treatment.

## Results

In group 1, 9 patients presented with Class I malocclusion and 15 subjects presented with Class II malocclusion. In group 2, Class I and Class II malocclusions were evenly distributed with 13 patients each. Upper arch dental crowding averaged 4.14±0.63mm and 3.03±1.02mm in groups 1 and 2; respectively (*P*<0.001).

Means, standard deviations (SD), mean differences, standard error (SE) and *P* values for the studied variables are shown in  Table 3- Table 5.

**Table 3:**
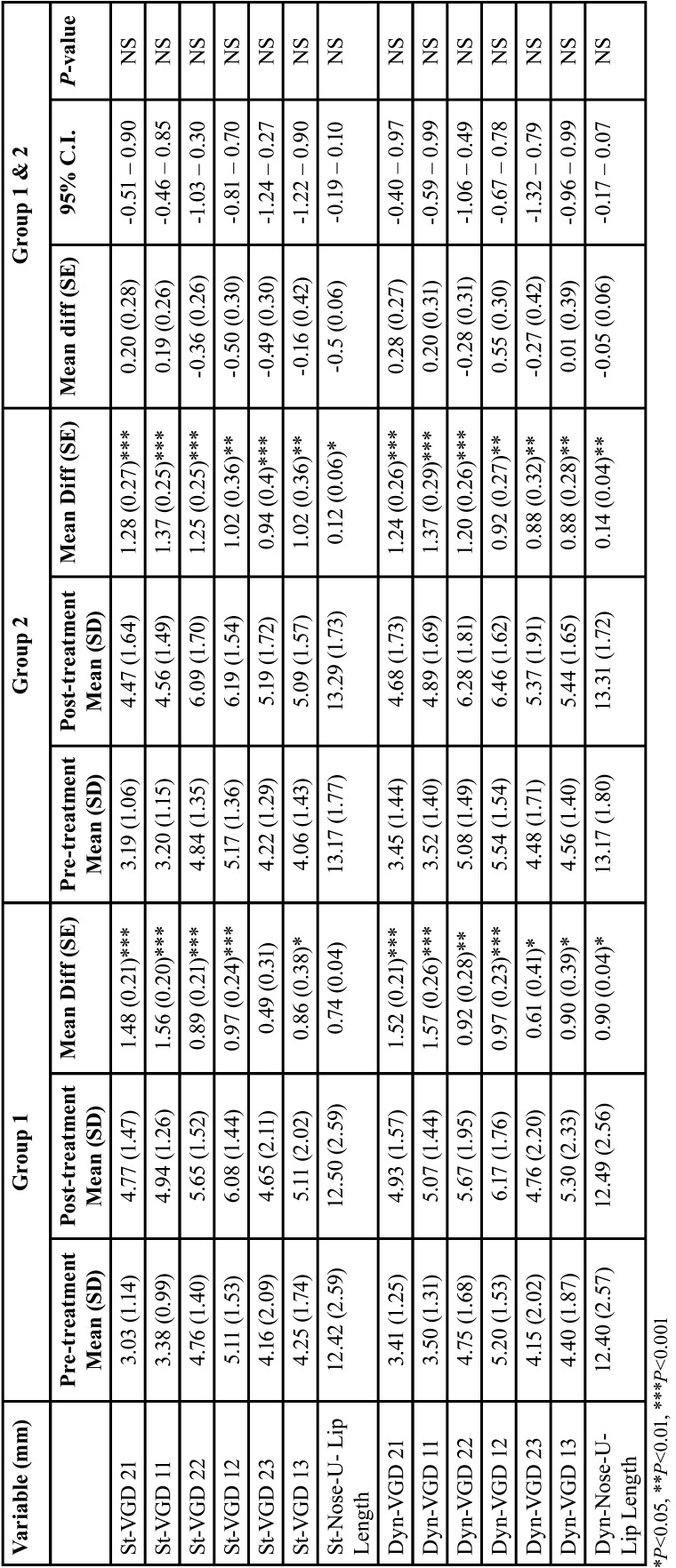
Means, standard deviations (SD), mean differences, standard error (SE), 95% confidence interval (C.I.) and P values for pre- and post- treatment VGD and lip length in static and dynamic photograph in the 2 studied groups.

**Table 4:**
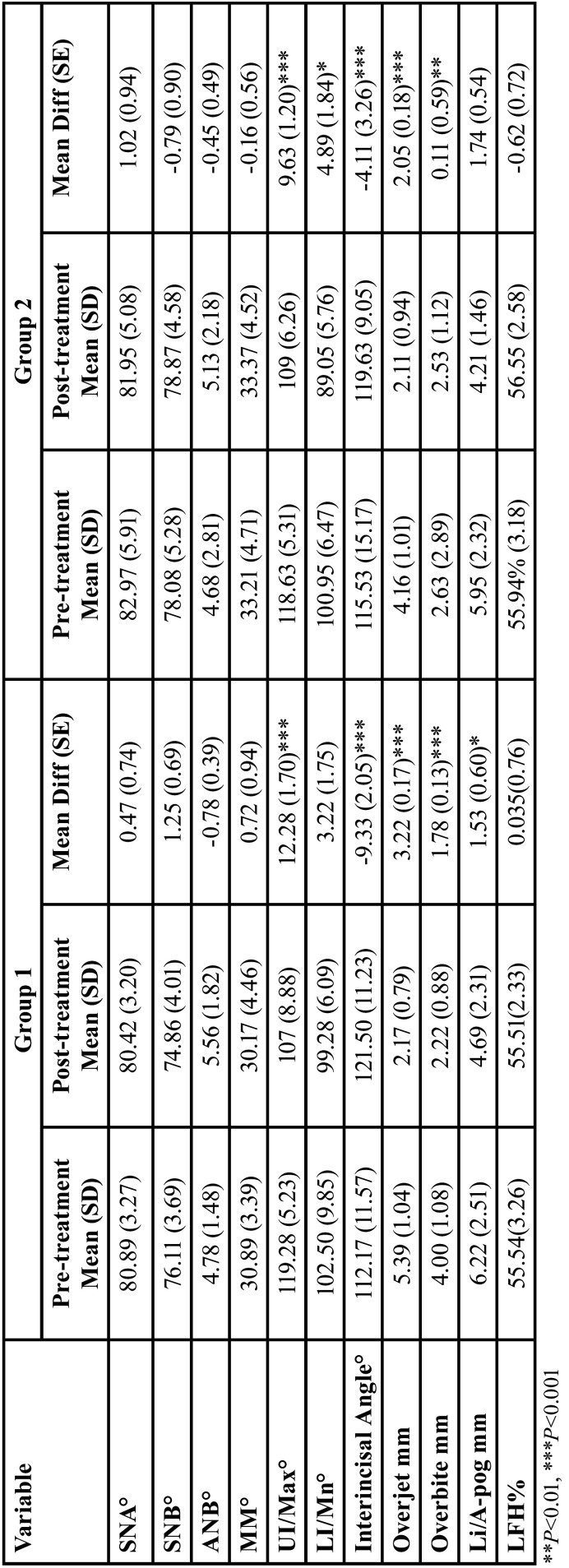
Means, standard deviations (SD), mean differences, standard error (SE) and P values for pre- and post- treatment cephalometric analysis in treated groups.

**Table 5:**
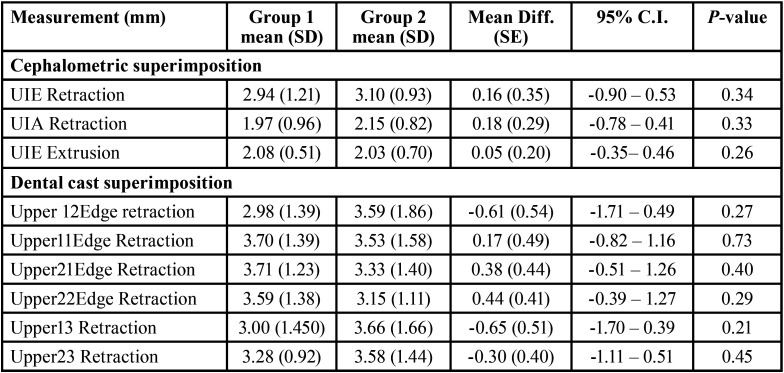
Means, standard deviations (SD), differences between the means of cephalometric and dental casts superimposition measurements, standard error (SE) and P-values in the studied groups.

In both bicuspid extraction groups, the differences between the pre- and post-treatment means for anterior VGD in the static and dynamic positions ranged from 0.61mm to 1.57mm which was statistically significant (*P*<0.05). The length of the upper lip increased after orthodontic treatment in both static and dynamic positions (*P*<0.05).

Significant changes were detected in the following cephalometric variables in both treatment groups; retroclined upper incisors (*P*<0.001), increased interincisal angle (*P*≤0.001), reduced overjet (*P*<0.001) and overbite corrected (*P*<0.01).

In both bicuspid extraction groups, the VGD increased in the static and dynamic positions. However, difference between the 2 groups was not statistically significant (*P*>0.05). There were no statistical differences detected in the amount of upper incisors edge and root retraction between the two extraction groups (*P*>0.05). In both bicuspid extraction groups, the amount of upper incisor extrusion during treatment was approximately 2mm (*P*>0.05).

Regression analysis showed that there were three predictors associated with the increase in the amount of VGD after upper premolars extraction (R=0.677); the amount of canine retraction (R2=0.498;*P*=0.001), the pretreatment ANB angle (R2=0.377;*P*=0.009) and the amount of upper incisor retraction (R2=0.301;*P*=0.030).

-Harms

No negative outcomes were reported by any subject during the trial. Gingival hyperplasia was observed in 2 cases and were excluded from the trial.

## Discussion

This study aimed to record the changes in the VGD associated with upper premolars extraction as part of orthodontic treatment plan. If upper premolars extraction is associated increased VGD, then upper teeth intrusion mechanics should be initiated as early as possible during orthodontic treatment. To our knowledge, this study was the first to investigate such changes in orthodontic subjects.

The age of included subjects ranged from 17 to 26 years to preclude growth changes; as growth of the upper lip could affect the gingival exposure measurements. Nanda *et al.* (9) reported that growth of the upper lip is usually completed by the age of 15 years in both males and females.

Although both genders were included in the study, there was a lower number of male subjects in all groups. This was in agreement with previous studies that reported higher demand for orthodontic treatment in females than in males (10). Additionally, smile patterns show sexual dimorphism. Previous findings agreed that GS is primarily a female characteristic and a higher smile line is more common in females than males (1).

 Patients were treated with fixed appliance with extraction of either upper first or second premolars according to the underlying orthodontic problem. Subjects who needed more space were treated with upper first bicuspid extraction, while patients who needed less space because they had moderate crowding were treated with upper second bicuspid extractions (11). The proper selection of teeth extraction based on the needed space resulted in comparable amount of anterior teeth retraction for both extraction groups. This would explain the comparable increase of VGD in both extraction groups after orthodontic treatment.

Space closure was carried on with elastic power chains on a rigid rectangular SS 0.019X0.025-inch archwire for all patients in order to achieve the maximum amount of bodily movement retraction of anterior teeth rather than tipping of the anterior teeth (12). However, tipping of teeth during retraction was evident as upper incisors inclination to maxillary plane was reduced significantly. This was likely due to the play between the archwire and bracket slot.

The amount of VGD was increased on the upper anterior teeth in both static and dynamic positions after premolars extraction. This increase in VGD may have resulted from anterior teeth extrusion during their retraction. This was in agreement with Sarver (13) who reported that space closure by uprighting the incisors through retraction would elongate the crowns of teeth which would in turn increase the amount of incisal show at rest and on smile.

Upper incisors retraction (≈3mm) and extrusion (≈2mm) after premolars extraction resulted in up to 1.5mm increase in VGD anteriorly. It has been suggested that because the gingiva and alveolus are attached to teeth roots by the periodontal ligament, the gingiva follows vertical movement of the root during extrusion forces (14). This explains the increase in VGD associated with upper anterior teeth retraction.

Lip length slightly increased after upper first and second bicuspid extraction which is in agreement with Janson *et al.* (15). Upper lip support may have been affected by the retraction of the upper anterior teeth which allowed the lip to achieve a lower position and thus increased the lip length (16). This finding may allow us to think that the increase in the VGD was slightly masked by the small increase in lip height.

The average anteroposterior changes in the position of the maxillary incisors found in this study were higher than those reported in previous studies (11-17). In this study, there was a mean maxillary incisor retraction of 3mm in both first and second bicuspid extraction groups. Ong and Wood (11) reported a mean maxillary incisor retraction of 2.5mm and 1.6mm in the maxillary first and maxillary second premolars extraction groups, respectively. Saelens and De Smit (17) reported an average 2.1mm and 1.9mm retraction in their 4 first bicuspids and 4 second bicuspids extraction groups, respectively.

Predictors of the increase in the amount of VGD after upper premolars extraction are the amount of canine and upper incisors retraction. This is of note to orthodontists where the greater the incisors and canine are retracted, the more the increase in the VGD. The pre-treatment ANB was also detected as a predictor to develop an increase in the amount of VGD after bicuspid extraction. The interpretation is that patients with a Class II malocclusion have a higher tendency to develop an increase in the VGD compared to a Class I malocclusion.

The limitations of this study include small male-to-female ratio. More males should be included in future studies. Also, the allocation of subjects in each group (first or second premolars) was not randomized and was based on the characteristics of the patients. In addition, skeletal Class I and Class II malocclusion was mixed in each group which might affected the outcome.

## Conclusions

- Extraction of both upper first and second premolars increases the amounts of the VGD in orthodontic patients 

- The increase in VGD following first and second premolars extraction was similar.

- The increase in VGD after orthodontic treatment is associated with the amount of canine retraction, pre-treatment ANB and the amount of incisor retraction.
